# H55N polymorphism is associated with low citrate synthase activity which regulates lipid metabolism in mouse muscle cells

**DOI:** 10.1371/journal.pone.0185789

**Published:** 2017-11-02

**Authors:** Brendan M. Gabriel, Mustafa Al-Tarrah, Yosra Alhindi, Audrius Kilikevicius, Tomas Venckunas, Stuart R. Gray, Arimantas Lionikas, Aivaras Ratkevicius

**Affiliations:** 1 School of Medicine, Medical Sciences and Nutrition, University of Aberdeen, Aberdeen, Scotland, United Kingdom; 2 Integrative Physiology, Department of Physiology and Pharmacology, Karolinska Institutet, Stockholm, Sweden; 3 Department of Applied Biology and Rehabilitation, Lithuanian Sports University, Kaunas, Lithuania; 4 Institute of Cardiovascular and Medical Sciences, University of Glasgow, Glasgow, Scotland, United Kingdom; Universidad Pablo de Olavide, SPAIN

## Abstract

The H55N polymorphism in the *Cs* gene of A/J mice has been linked to low activity of the enzyme in skeletal muscles. The aim of the study was to test this hypothesis and examine effects of low citrate synthase (CS) activity on palmitate metabolism in muscle cells. Results of the study showed that carriers of the wild type (WT) *Cs* (C57BL/6J and Balb/cByJ mouse strains) had higher CS activity (p < 0.01) than carriers of the A/J variant (B6.A-(rs3676616-D10Utsw1)/KjnB6 and A/J mouse strains) in the heart, liver and gastrocnemius muscle. Furthermore, the recombinant CS protein of WT showed higher CS activity than the A/J variant. In C2C12 muscle cells the shRNA mediated 47% knockdown of CS activity reduced the rate of fatty acid oxidation compared to the control cells. In summary, our results are consistent with the hypothesis that H55N substitution causes a reduction in CS activity. Furthermore, low CS activity interferes with metabolic flexibility of muscle cells.

## Introduction

Mitochondria play a key role in metabolic health and functioning of skeletal muscle [1,2]. Impairments in mitochondrial function are often associated with degenerative diseases [3]. Citrate synthase (CS) is a key enzyme of the mitochondrial Krebs cycle and its activity is often used as a biomarker of mitochondrial content and function [4]. Indeed, improvements in whole body oxidative capacity after endurance training are matched by increases in muscle CS activity in a nearly 1:1 relationship [5]. Interestingly, however, the maximal capacity of CS to produce citrate exceeds the rate of use in the Krebs cycle by more than 10 times, even when assessed in skeletal muscles during exercise at a maximal aerobic capacity [6]. In agreement with these calculations, cytosolic citrate levels increase significantly in skeletal muscles of rats after glucose infusion which increases substrate availability for citrate synthesis in mitochondria [7]. These findings suggest that CS activity might be higher than needed to sustain mitochondrial function.

We have observed that A/J mice show only about half the CS activity in the quadriceps and gastrocnemius muscles compared to other five strains of mice [8]. This low CS activity was not due to either reduced mitochondrial or CS protein content and we have attributed it to a missense mutation, in exon 3 of *Cs* gene of A/J mice, i.e. the H55N substitution (A for C, rs29358506) which is predicted to affect protein function [8]. Variations in CS activity might be of importance for functioning of cells though little is known about the associated mechanisms. Johnson and colleagues [9] have linked the H55N polymorphism to hearing loss in the congenic B6.A-(rs3676616-D10Utsw1)/KjnB6 (B6.A) mice which have the C57BL/6J (B6) strain background, but carry the A/J allele in the telomeric region of chromosome 10 harbouring the *Cs* gene. It has also been suggested that innate impairment of CS functioning is responsible for low rates of fatty acid oxidation and thus triggers insulin resistance in muscle fibres of diabetic patients [10,11]. Thus variation in CS activity might be of importance for functioning of cells, though little evidence is provided in support of this hypothesis.

In previous studies we have only assessed CS enzyme activity in samples of the skeletal muscle [8,12]. CS is encoded by a single gene and its enzyme activity should, therefore, be reduced in all tissues which contain mitochondria in A/J mice. So far, we were unable to find clear experimental evidence to support or reject this contention. Furthermore, the causative link between H55N substitution and CS activity has not been validated. Although CS activity can be suppressed to negligible levels by a mutation in the oxaloacetate binding site [13], H55N substitution occurs in the vicinity of the CS active site and its effects require experimental testing.

The first aim of the current study was to compare CS activity in the gastrocnemius muscle, heart and liver of C57BL/6J (B6), Balb/cByJ (BALB), A/J, B6.A and the offspring of the cross between B6 and B6.A (B6/B6.A) mice. We hypothesised that carriers of the A/J variant of H55N polymorphism (A/J and B6.A strains) would show lower CS activity compared to the B6 and BALB strains which carry the wild type (WT) of the *Cs* allele. Our second aim was to test the causative link between the H55N polymorphism and CS activity. We overexpressed and purified CS protein using the B6 or A/J strain constructs of Cs and compared their enzymatic activity. Finally, we assessed physiological effects of shRNA mediated knock down of *Cs* expression, which resulted in reduction of CS activity analogous to H55N polymorphism in terms of magnitude. Our results show that the A/J allele of the Cs gene conferred low CS enzyme activity in all the studied tissues. The recombinant CS protein showed lower enzyme activity when it was generated using A/J construct rather than B6 construct. The shRNA mediated knockdown of *Cs* expression led to a reduction in the rate of fatty acid oxidation and increased accumulation of ceramide in C2C12 muscle cells. These findings confirm the importance of CS activity in cellular metabolism, especially during the exposure to high concentrations of saturated long chain fatty acids, such as palmitate.

## Materials and methods

All reagents were from Sigma-Aldrich (Poole, United Kingdom) unless indicated otherwise.

### Animals

The study was approved by the animal research ethics committee at the Lithuanian State Food and Veterinary Service (approval No. 0223). This included euthanasia of animals by cervical dislocation at 14–15 weeks of age. All procedures were similar to those previously described [8]. Mice represented the following strains: BALB/cByJ (BALB, n = 6), B6 (n = 13), B6.A-(rs3676616-D10Utsw1)/KjnB6 (B6.A) (n = 13), offspring of the cross between B6 and B6.A, F1 (n = 9) and A/J (n = 6). The congenic strain, B6.A, carries the A/J allele between rs3676616 and D10Utsw1 markers which flank *Cs* gene on chromosome 10 on otherwise B6 strain background [9]. The animals were kept at a temperature of ~22°C at 40–60% humidity, fed chow diet and received tap water ad libitum.

### Tissue sampling

Immediately after euthanasia, livers, hearts and gastrocnemius muscles were excised and snap frozen in isopentane pre-cooled by liquid nitrogen and stored at -80°C. For protein analysis the samples were weighed (Electronic balance KERN ABS 80–4, Germany) and homogenised in ice cold lysis buffer (50 mM Tris-HCl, 1 mM EDTA, 1 mM EGTA, 1% (vol/vol) Triton X-100, pH was adjusted to 7.5) using an ULTRA–TURRAX homogeniser (Rose Scientific, Edmonton, Canada). The homogenates were shaken for 60 min at 4°C and then centrifuged at 13,000 *g*, 4°C for 10 min. The supernatants were taken for spectrophotometric measurements (GENESYS 10 Bio UV-Vis Spectrophotometer; Thermo Fisher Scientific Inc., Waltham, MA, USA) of the protein concentration (Bradford assay, Bio-rad, Hertfordshire, UK) and CS enzyme activity.

### Purification of recombinant citrate synthase (CS) protein

For B6 protein, the cDNA corresponding to the B6 strain variant of *Cs* was obtained in an expression vector EX-Mm01963-B01 (p-Receiver-B01 vector, T7 promoter, N-His tag, GeneCopoeia) and amplified by transformation into competent E. coli cells of XL1-blue strain (Agilent). DNA was then extracted using Endo-free plasmid purification kit (Qiagen, Crawley, UK). For A/J protein, the Stratagene QuikChange™ site-directed mutagenesis kit and primer pair, 5’-GACCTTCAAGCAGCAAAATGGGAAGACAGTGG-3’ and 5’-CCACTGTCTTCCCATTTTGCTGCTTGAAGGTC-3’, were used to introduce H55N substitution, A for C at rs29358506 [8]. The result of this transformation was verified by sequencing and genotyping. For genotyping PCR was performed using the primer pair, TACCTAAGGAGCAGGCCAGA and CAGCCAAAATAAGCCCTCAG, and the product digested using Fat1 (New England Biolabs). The digest was performed for 1 h at 37°C and the products were separated using 3% agarose gel. For production of the recombinant CS protein, standard procedures were followed [14]. The plasmids were transformed into RosettaBlue (DE3) cells (Novagen) and induction of the His-tagged polypeptides was triggered by 1 mM isopropyl β-Δ-thiogalactoside (IPTG) which was added for 0.5, 1, 2, 3, 6, 12, 18 or 24 h at 37°C when bacterial cultures reached the optical density of 0.5–0.7 at 600 nm wavelength. The bacterial cultures were grown in Luria-Bertani media (10 g/l tryptone, 5 g/l yeast extract, and 10 g/l NaCl) containing 0.0125 g/l tetracyclin and 0.043 g/l chloramphenicol. Cells were harvested by centrifugation at 3000 g, 4°C for 10 min. Cell pellets were lysed by freeze thawing and incubation with 0.5 mg/ml lysozyme at 4°C and centrifuged at 10,000 *g*. Recombinant His-tagged CS proteins were purified from the soluble fraction using Ni-NTA resin (QIAGEN, Crawley, West Sussex, UK) at 4°C. Initially, the resin was pre-washed using low salt buffer (20 mM Tris-HCl, 500mM NaCl, 5% glycerol, 5mM imidazole pH 7.9). After application of the cell lysate the resin was washed again with the same low salt buffer to remove the unbound proteins. Finally, the bound proteins were eluted from the resin with the high salt buffer (20mM Tris-HCl, 500 mM NaCl, 5% glycerol, 200 mM imidazole pH 7.9) and collected in 1.5 ml fractions. The presence of CS protein was determined using immunoblotting and CS assay. See sections “Immunoblotting” and “Mitochondrial enzymes and citrate” for details about these methods.

### C2C12 muscle cell culture

C2C12 mouse muscle cells were used to study effect of reduced CS activity on cellular metabolism. As previously described [15], mouse C2C12 muscle cells were cultured in growth (G) medium containing 88% (vol/vol) Dulbecco’s Modified Eagle’s medium (DMEM), 20 mM glucose, 10% (vol/vol) FCS and 2 mM glutamine in T75 cm^2^ flasks at 37°C and 5% CO_2_. For all experiments, ~2 x 10^6^ cells were seeded in 6-well tissue culture plates coated with extracellular matrix gel (E6909, Sigma-Aldrich, MO, USA) and containing 2 ml of the G medium. When cells became confluent, the medium was changed to the differentiation (D) medium containing 96% DMEM, 20 mM glucose, 2% horse serum and 2 mM glutamine. The medium was changed every day for 5 days to allow cells to differentiate for metabolic and or other measurements.

### Citrate synthase (CS) knock down

Lentivirus delivered stable gene silencing was used to knock down *Cs* expression in C2C12 muscle cells using similar methods as in other studies [16]. Pseudoviruses were produced by co-transfecting HEK293 cells with a plasmid carrying shRNA and the Mission lentiviral packaging mix (SH001, Sigma–Aldrich) containing plasmids expressing viral packaging genes and a heterologous viral envelope gene. We used shRNA against Cs mRNA, i.e. GCACCCAACATTTGAGTTATTCTCGAGAATAACTCAAATGTTGGGTGC which targets the 3’untranslated region (UTR) of Cs mRNA (Cs shRNA) and control shRNA (Con shRNA) containing scrambled shRNA sequence. The shRNA were delivered within the pLKO.1-puro vector containing the puromycin resistance marker. Virus was harvested in the culture supernatant at 72 h post-transfection and transductions of C2C12 cells at low confluence were carried out in the presence of 10 μg/ml of polybrene. After transduction, cells were selected in 3 μg/ml of puromycin for 3 days before being used in experiments. C2C12 cells treated with Con shRNA and Cs shRNA are referred to as Con shRNA and Cs RNA cells, respectively, in the manuscript.

### Cell growth

A cell impedance assay was carried out on Con shRNA and Cs shRNA cells. ~1500 cells were seeded in 96-well plate which was inserted into a cell analyser (xCELLigence System, Roche). xCELLigence analyser monitors cellular events in real time by measuring electrical impedance across interdigitated micro-electrodes integrated on the bottom of the well plates. This electrical impedance is referred to as Cell index which depends on cell number, adhesion, viability and morphology.

### Cell metabolism

A Seahorse Bioscience XF24-3 Extracellular Flux Analyser was used to measure changes in the dissolved O_2_ and pH in the medium surrounding C2C12 cells cultured in XF24-well microplates (Seahorse Bioscience). Initially, wells (0.32 cm^2^) were coated with the extracellular matrix gel. Then cells were seeded at ~12,500 cells per well in 250 ml of G medium. After 2 days, the media was changed to D media and cells were allowed to differentiate and form myotubes. For assessment of fatty acid oxidation, cells were initially incubated in substrate limited medium DMEM (0.5 mM glucose, 1 mM glutamine, 0.5 mM carnitine and 1% fetal bovine serum) for 24 h before the experiment. Then the medium was changed to 560 μl of fatty acid oxidation medium (111 mM NaCl. 4.7 mM KCl, 1.25 mM CaCl2, 2 mM MgSO_4_, 1.2 mM NaH_2_PO_4_) and cells were left in the incubator for 60 min at 37°C without CO_2_. Afterwards XF24-well microplate was transferred to Seahorse Bioscience XF24-3 Extracellular Flux Analyser and measurements of oxygen consumption rate (OCR) and proton production rate (PPR) were started. After evaluation of basal metabolism, solutions of inhibitors and uncouples were injected into the cell media one after another. Firstly, oligomyocin concentration was adjusted to 2.5 μM in order to inhibit aerobic ATP synthesis in mitochondria. This was followed by injection of Carbonyl cyanide-4-(trifluoromethoxy) phenylhydrazone (FCCP) to generate 1.6 μM FCCP which would induce maximal mitochondrial respiration by uncoupling aerobic ATP synthesis from the electron transport chain. Then concentrations of rotenone and antimycin A were adjusted to 4 μM and 2 μM, respectively, in order to inhibit electron transport chain by targeting NADH dehydrogenase and cytochrome c reductase, respectively. Finally, cells were lysed for determination of protein content using Bradford assay. As in other studies [17], the following parameters were then calculated: basal respiration (initial respiration before injection of oligomycin), maximal respiration (maximal uncoupled respiration as induced by FCCP minus non-mitochondrial respiration as measured after application of rotenone and antimycin A), spare respiratory capacity (maximal uncoupled respiration minus basal respiration), proton leak (respiration prior to FCCP injection minus non-mitochondrial injection), and ATP production (basal respiration minus proton leak).

### Palmitate metabolism

For assessment of palmitate metabolism C2C12 cells were incubated in the media containing 20 mM Hepes, 140 mM NaCl, 16.1 mM KCl, 5.1 mM MgSO_4_, 2.7 mM CaCl2, 1.2 mM l-carnitine; 2% fatty-acid-free BSA, 0.8 mM palmitate and 2 μCi [^14^C]palmitate (PerkinElmer—NEC534050UC) with pH adjusted to 7.4. In the second series of experiments with mixed substrate 5.5 mM glucose was added to the media. Following 2-h incubation period, 1 ml of the medium was transferred to 15 ml tube, the cap of which housed a Whatman (GF/B) filter paper disc that had been pre-soaked with 200 μl of 1M KOH. [^14^C]O_2_ trapped in the media was then released by acidification of media using 60% (vol/vol) perchloric acid and gentle agitation of the tubes at 37°C for 2 h. Afterwards, the filter paper disc was removed from the cap and placed into 2 ml of scintillation liquid. The remaining cells were subsequently washed with 2 ml of phosphate buffered saline and lysed in 1 ml of 1 M NaOH before being transferred into 4 ml of scintillation liquid. Samples were then subjected to liquid scintillation counting (Wallac 1409 Liquid Scintillation counter, PerkinElmer, Waltham, MA, USA). The protein content of samples was measured in triplicates using Bradford assay.

### Ceramide immunohistochemistry

Immunohistochemistry was applied for assessment of changes in cellular levels of ceramide. C2C12 muscle cells were grown and differentiated on extracellular matrix coated 96 well plates. Prior to initiating the assessment cells were starved in substrate limited medium (0.5 mM glucose, 1 mM glutamine, 0.5 mM carnitine and 1% FBS) for 24 h. The cells were then incubated in substrate enriched media, i.e. DMEM supplemented with 0.8 mM palmitate, 0.5 mM carnitine, 100 nM insulin with 5.5 mM glucose (P+G experiments) or without it (P experiments) for 3 h, 12 h and 18 h. Afterwards, C2C12 muscle cells were fixed for 10 min with 4% formaldehyde and permeabilized with 0.4% Triton X-100 in PBS for 6 min, washed, and blocked by 20% goat serum (Dako, UK) in PBS for 30 min. Then monoclonal antibody against ceramide (C8104, Sigma Aldrich, UK) was diluted at 1:10 in 0.025% Tween 20 in PBS solution and applied onto cells with gentle agitation on a rocker for 24 h at 4°C. Afterwards, the secondary antibody anti-mouse coupled to Alexa fluor 555 (Abcam, UK) diluted at 1:500 in 0.025% Tween 20 solution was applied for 90 min. To stain nuclear DNA, C2C12 muscle cells were incubated in 4,6’-diamidino-2-phenylindole (DAPI) (Thermofisher, UK) at 1:1000 in PBS for 30 min covered from light on a rocker. C2C12 muscle cells were observed using Zeiss observer Z1 imaging confocal system (Carl Zeiss, Germany) and the images were captured using Axiocam (Carl Zeiss, Germany). Fluorescence was quantified between treatments as a single in focus plane was acquired. Using ImageJ (v1.49) a single coloured image was split into grey/black images from their red and blue fluorescence and an outline was drawn around each grey area and the mean fluorescence was measured, along with black background readings. The total corrected cellular fluorescence (TCCF) = integrated density–(area of selected cells × mean fluorescence of background readings), was calculated. Ceramide mean TCCF was then normalised against the mean TCCF of nuclear DNA in the same field of view.

### Real time RT-PCR

Cell samples were homogenised in 1 ml of ice cold TRIZOL Reagent (Invitrogen Ltd, Paisley, UK) and RNA extracted using chloroform and isopropanol as described previously [8]. 2 μg of RNA was then used for cDNA synthesis in 20-μl reaction volume containing 50 mM Tris-HCl (pH 8.3), 75 mM KCl, 3 mM MgCl_2_, 0.5 mM dNTP Mix (0.5 mM each dATP, dGTP, dCTP and dTTP), 5 mM DTT, 150 ng of Random primers and 200 units of SuperScript™ III Reverse Transcriptase. Real time PCR was performed using Roche Lightcycler 480 II (Roche Diagnostics, Sussex, UK) and Multiplex Taqman assays for *Cs* as a target gene and *β-Actin* as a reference gene in each sample. Three μL of cDNA was added to 10 μL of LightCycler^®^ 480 Probe Master (Roche), 0.2 μL of TaqMan^®^ probe (Probe no. 100, Universal Probe Library), 0.2 μL of forward and reverse primers (20 μM) each and 1 μL of mouse β-Actin probe dye VIC-MGB (Applied Biosystems, 4326317E). The mouse *Cs* intron spanning primers were designed using Universal Probe library software and purchased from Sigma-GenoSys (Forward primer: 5’-GGAAGGCTAAGAACCCTTGG-3’ and Reverse primer: 5’-TCATCTCCGTCATGCCATAGT-3’) and the corresponding UPL probe (UPL probe #100) were used. The results were analysed using LightCycler^®^ 480 software 1.5 and *Cs* was normalised to β-Actin and presented as a ratio (Ratio = (1 + E_Cs_)^**-Ct[*Cs*]**^ / (1 + E_*βactin*_)^**-Ct[*β-actin*]**^) [18].

### Immunoblotting

As previously described[8], cell and tissue samples were homogenised in ice cold lysis buffer (50 mM Tris-HCl, 1 mM EDTA, 1 mM EGTA, 1% (vol/vol) Triton X-100, 0.1% (vol/vol) 2-mercaptoethanol, pH = 7.5) supplemented with protease inhibitor cocktail, 10 mM β-glycerophosphate, 50 mM NaF and 0.5 mM Na_3_VO_4_. Protein concentration was determined in triplicate using the Bradford Assay (Bio-rad, Hertfordshire, UK). For immunoblotting, the samples were loaded on 10% polyacrylamide gel, separated using SDS-PAGE and then transferred to polyvinylidene fluoride (PVDF) membrane. Then membranes were washed with Tris buffered saline (TBS) containing 0.1% (vol/vol) Tween-20 (TBS-T buffer) before being blocked with 5% (wt/vol) non-fat milk in TBS-T buffer. For analysis of proteins carrying His tags, membranes were incubated for 3 h at 4°C with the anti-polyHistidine antibody (1:4000 dilution, H1029, Sigma-Aldrich, UK). For CS protein analysis, membranes were incubated overnight with the CS antibody (1:1000 dilution, #CIS11-A, Alpha Diagnostic, San Antonio, TX, USA). Analysis of cell signalling was performed using the overnight incubation antibodies from Cell Signalling Technology (Danvers, MA, USA). The primary antibodies against AMPK (#2532), phospho-AMPKα (Thr172) (#2531), ACC (#3662), phospho-ACC (ser79) (#3661), β-actin (#4967), p44/44 MAPK (#9102), phospo-p44/42 MAPK (Thr202/Tyr204) (#9101), p38 MAPK (#9212), phospo-p38 MAPK (Thr180/Tyr182), mTOR (#2972) and phospo-mTOR (Ser2448) (#2971). The exposure to the primary antibodies was followed by 2-h incubation with HRP-conjugated secondary antibody (1:2000 dilution, #7071, Cell Signalling, Beverly, MA, USA) and detection using ECL detection reagent (Amersham Biosciences, Buckinghamshire, UK) and Fluor-SMax Imager for immunobloting (Bio-rad, Hertfordshire, UK). The images were quantified using ImageJ (NIH, USA) software.

### Mitochondrial enzymes and citrate

CS activity was measured as previously described [8]. The reaction reagent consisted of 100 mM triethanolamine-HCl, DTNB (100 µM), 0.25% Triton-X (vol/vol), 0.5 mM oxaloacetate, 0.31 mM acetyl CoA with pH adjusted to 8.0. Ten μl of muscle homogenate was added to start the reaction in 1000 μl. The molar extinction coefficient of 13,600 M^–1^·cm^–1^ was used to assess the maximum CS activity (V_max_) at 412 nm during the first 2 min of the reaction. The assay was carried out at room temperature (~21°C), and CS from porcine heart was used as a standard (C3260-200UN, Sigma-Aldrich, UK) for assay calibration. For β-hydroxyacyl-coenzyme (CoA) dehydrogenase (HAD) assay, the molar extinction coefficient used was 63,000 M^-1^cm^-1^ for NADH at 340 nm. The reaction reagent consisted of 100 mM tetrasodiumpyrophosphate, 0.23 mM NADH, 0.24 mM acetoacetyl CoA with pH adjusted to 7.3. The 1000 μL of reaction reagent included 20 μL of muscle homogenate. Citrate concentration was determined using the standard colorimetric-enzyme assay kit (MAK057, Sigma-Aldrich) and the plate reader (Synergy HT, Biotek, VT, United States). For sample preparation, cells were lysed, homogenized and centrifuged at 13,000 *g* for 20 min using 10 kDa molecular weight cut off filters (Amicon Ultra, Millipore, MA, USA) to generate filtrates for citrate measurements. For cytochrome C oxidase (COX) measurements, the pellets from the filters were removed by centrifugation at 10,000 *g* for 10 min and re-constituted in 300 μl enzyme dilution buffer of standard COX assay kit (CYTOCOX1-1KT, Sigma-Aldrich). The protein concentration of was always assessed using Bradford assay.

### Statistical analysis

All data analysis was performed using Prism 3.0 software. Analysis of variance (ANOVA) followed by t-tests with Bonferroni correction for multiple comparisons were used to assess differences between the means.

## Results

**Fig 1:**
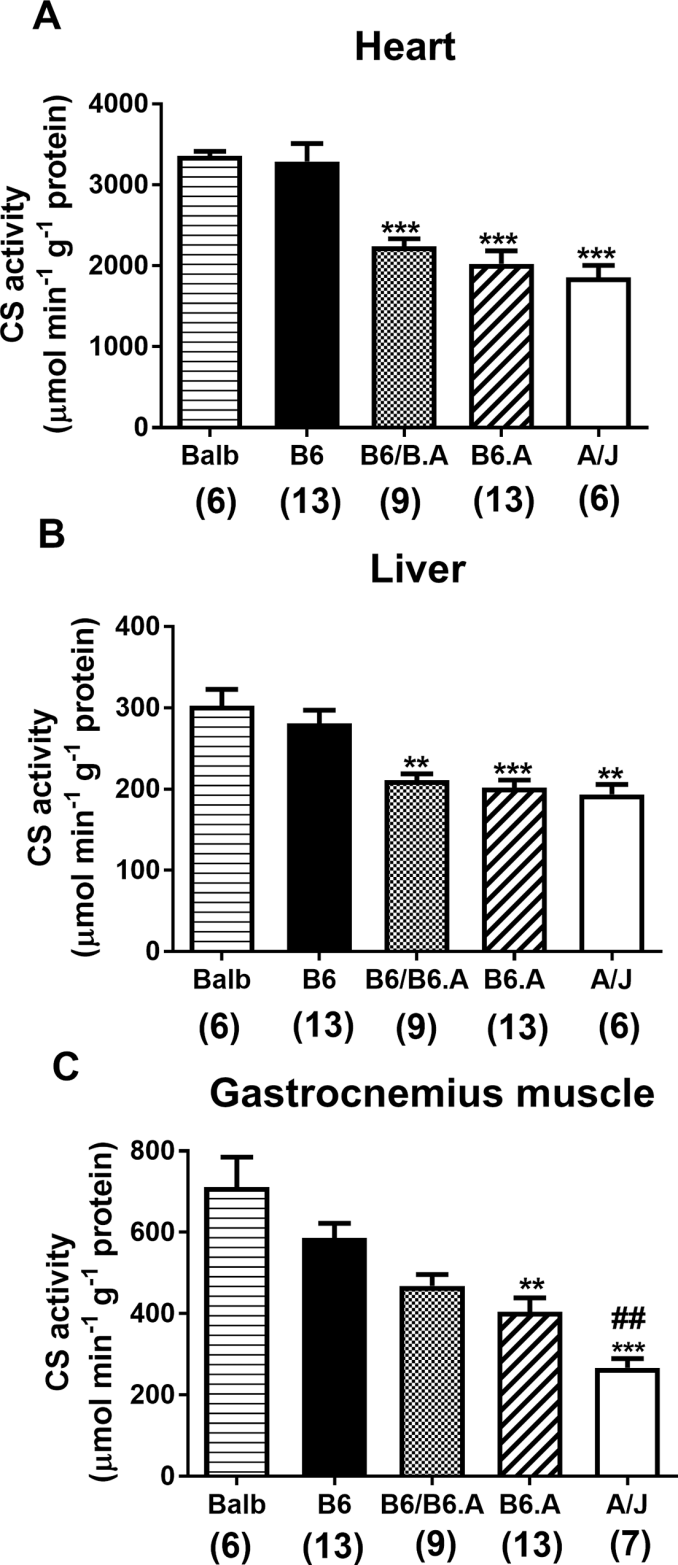
Citrate synthase (CS) activity in mouse tissues. Data are shown for the heart (A), liver (B) and gastrocnemius (C) muscle of BALB, C57BL/6J (B6), offspring of the cross between B6 and congenic B6.A-(rs3676616-D10Utsw1)/KjnB6 (B6/B6.A) mice, congenic B6.A and A/J mice, respectively. CS activity and protein levels were measured using spectrophotometric assays. Data are shown as mean ± S.E. Numbers of samples are indicated below in brackets. ** *p* < 0.01, *** *p* < 0.001 are different to BALB and B6 strains, respectively; ## *p* < 0.01 different to B6/B6.A mice, respectively.

Data on CS activity in the heart, liver and gastrocnemius muscle of the mice are presented in Fig 1. CS activity depended on the strain (*p* < 0.0001) in all the tissues. For the heart and liver, BALB and B6 mice showed consistently higher CS activity than B6/B6.A, B6.A and A/J mice. In the gastrocnemius muscle B6.A and A/J strains had lower CS activity than BALB and B6 strains, while CS activity for A/J strain was also lower (*p* < 0.05) compared to B6/B6.A mice.

**Fig 2:**
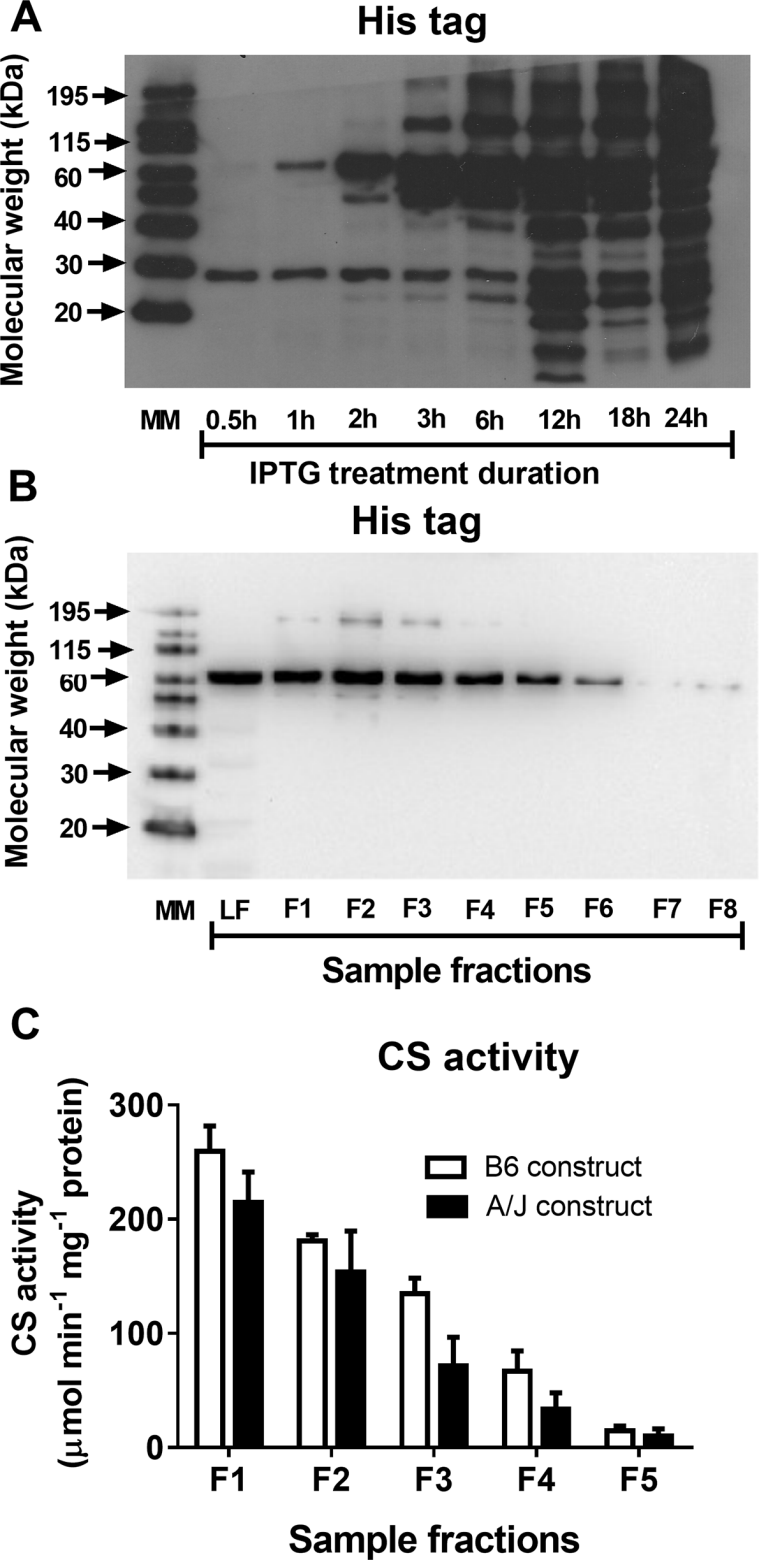
Overexpression and purification of citrate synthase (CS). A, Immunoblots of recombinant His tagged proteins in lysates of RosettaBlue (DE3) cells transformed with EX-Mm01963-B01 plasmid and exposed to 1 mM isopropyl β-Δ-thiogalactoside (IPTG) for different durations at 30 degrees C, B, Immunoblots of soluble His-tagged proteins from RosettaBlue (DE3) cells after purification with Ni-NTA resin. Different protein fractions (F) eluted from Ni-NTA resin by high salt buffer were tested. LF is the lost fraction that was collected just after the lysate was applied onto the resin before elution of the proteins using high salt buffer. Immunoblots are representative of four separate experiments with CS overexpression and purification. C, Citrate synthase activity in the first five fractions of proteins eluted from Ni-NTA resin using high salt buffer for B6 construct and A/J construct of Cs, respectively. Results are representative of four independent experiments. Data are shown as mean ± S.E.

Results from the experiments with purification of recombinant CS protein are presented in Fig 2. When treated with 1 mM IPTG for 2 h and longer the transformed RosettaBlue (DE3) cells showed a marked increase in content of His tagged proteins with a molecular weight of ~60 kDa, which corresponds to the molecular weight of CS (Fig 2A). We used 2 h exposure of the cells to IPTG for the purification of recombinant CS protein by applying to Ni-NTA resin. His-tagged proteins and CS activity were detected in sample fractions that were eluted from the resin using the high salt (200 mM imidazole) buffer (Fig 2B). The two-way ANOVA showed that CS activity decreased progressively with the increase in fraction number (*p* < 0.0001) and was higher (*p* < 0.05) for B6 construct compared to the A/J construct (Fig 2C).

**Fig 3:**
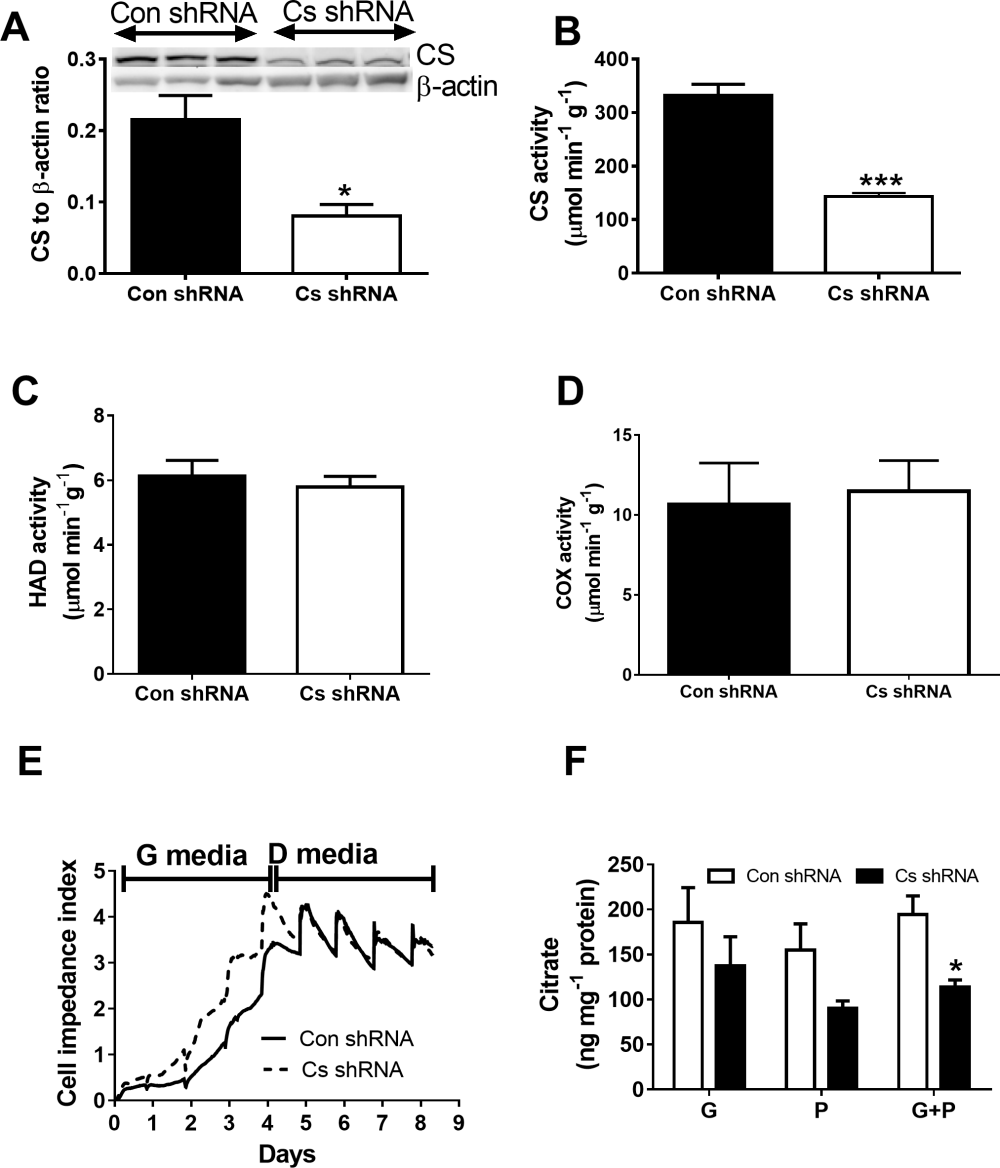
Citrate synthase (CS) activity, mitochondrial markers and proliferation of Con shRNA and Cs shRNA cells. A, citrate synthase (CS) activity; B, CS protein levels; C, HAD activity; D, cytochrome C oxidase (COX) activity; E, proliferation rate as reflected in cell impedance index of growing cells. CS and HAD activity (n = 9 each) was measured using a spectrophotometric assays. COX activity (n = 6) was assessed using COX assay kit; F, Citrate accumulation was measured using the standard assay kit. Values for CS, HAD, COX, and citrate are shown as mean ± SEM. Cell impedance index was assessed in cells incubated in growth media and differentiation media (n = 4). Cells were grown in 6 well plates and the media was changed every day.

Results demonstrating the effects of low CS activity on C2C12 mitochondrial markers and cell growth are presented in Fig 3. We used lentivirus delivered stable gene silencing to knock down *Cs* expression in C2C12 muscle cells and Cs mRNA to β-Actin mRNA ratio was consistently lower in *Cs* shRNA cells compared to Con shRNA cells (0.022 ± 007 versus 0.081 ± 016, respectively, *p* < 0.001). CS protein content was also halved and there was ~47% reduction (*p* < 0.001) in CS activity for *Cs* shRNA cells compared to Con shRNA cells (Fig 3A and 3B, respectively). Thus, the reduction in CS activity was of a similar magnitude to that of the H55N polymorphism induced reduction. There were no differences between these two types of cells in activity of mitochondrial HAD or COX (Fig 3C and 3D, respectively). CS knockdown was associated with a faster (*p* < 0.05) increase in cell index though peak values of this index did not differ between Cs shRNA and Con shRNA cells (Fig 3E). Citrate levels were higher (*p* < 0.05) in Con shRNA cells compared to Cs shRNA cells (Fig 3F).

**Fig 4:**
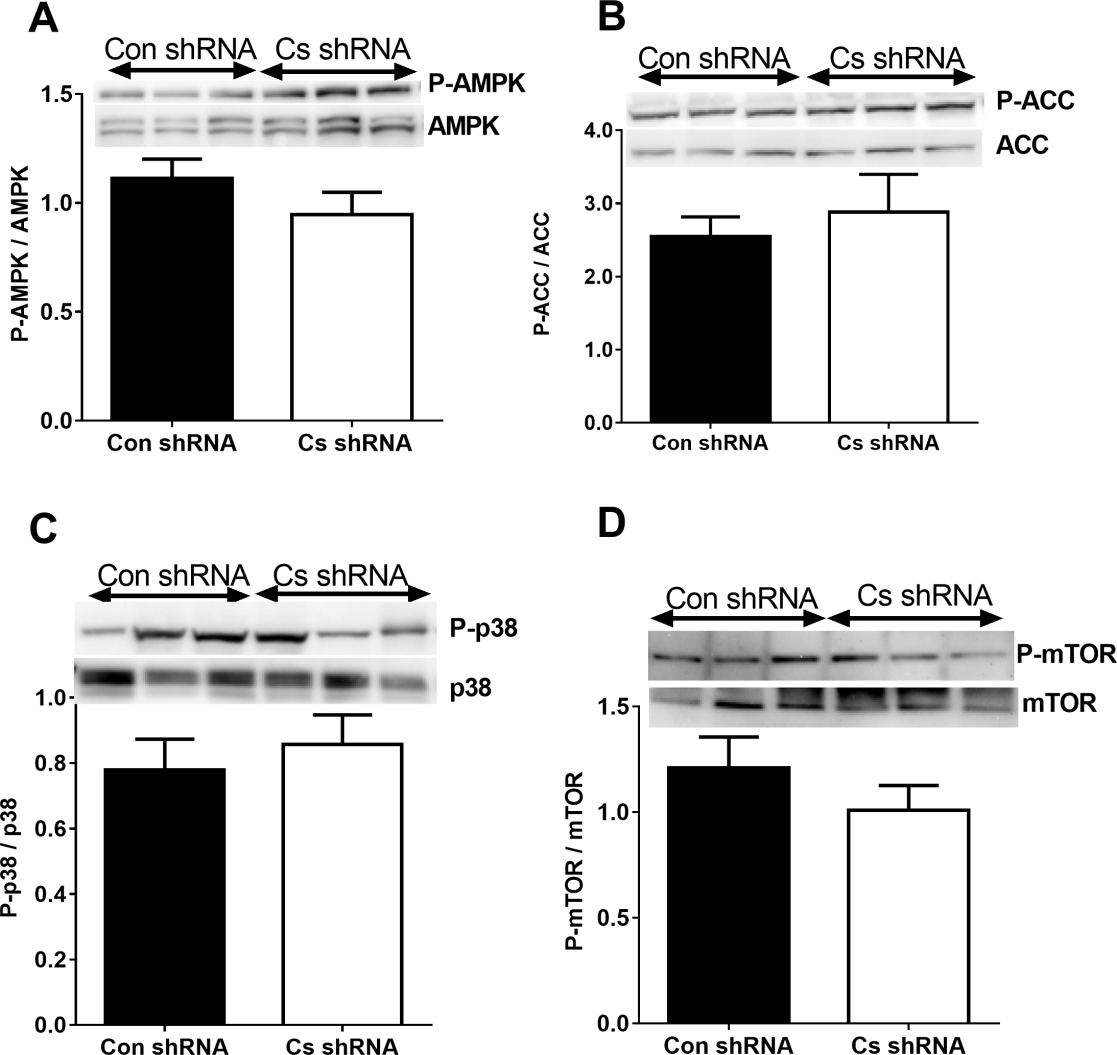
Cell signalling proteins in Con shRNA and Cs shRNA myotubes. The protein levels were was assayed by immunoblotting and the blots were analyzed using ImageJ software. The ratios of phosphorylated to total protein were calculated for AMPK (A) and ACC (B), MAPK p38 (C) and mTOR (D) for Con shRNA cells (n = 8) and Cs shRNA (n = 8) cells. Values are means ± SEM.

There were no differences between Cs shRNA and Con shRNA cells in phosphorylation of AMPK (Fig 4A), ACC (Fig 4B), MAPK p38 (Fig 4C) or mTOR proteins (Fig 4D).

**Fig 5:**
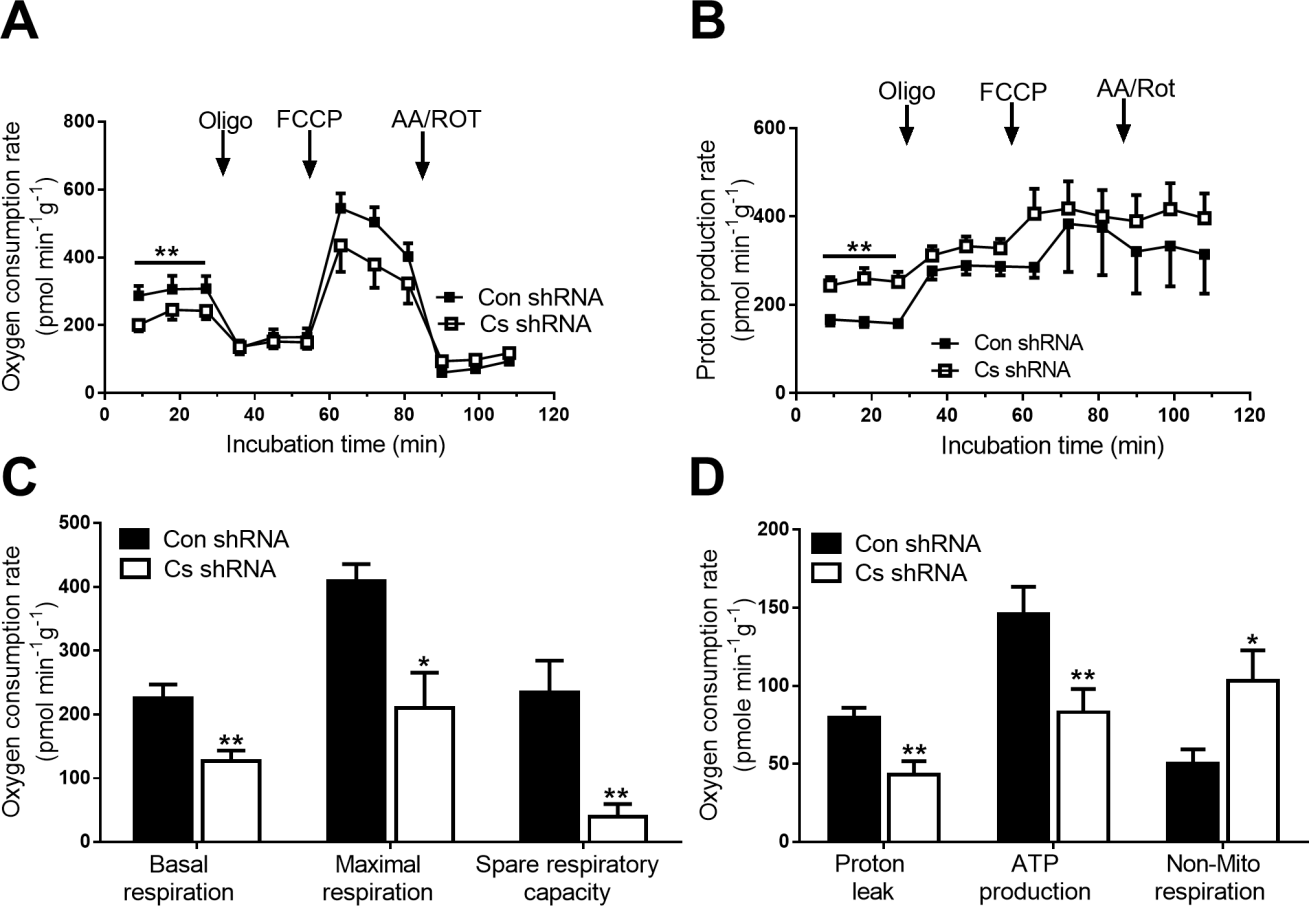
Cellular metabolism in Con shRNA and Cs shRNA myotubes. A, Oxygen consumption rate and (B) proton production rate were assessed using Seahorse Bioscience XF24-3 Extracellular Flux Analyser; C, Basal respiration, maximal respiration and spare respiratory capacity; D, Proton leak, aerobic ATP production and non-mitochondrial respiration. Values are means ± SEM (n = 10 each); *P < 0.05, ** *p* < 0.01 different between Con shRNA and Cs shRNA myotubes.

Oxygen consumption and proton production of Cs shRNA and Con shRNA cells was measured after 24 h period of starvation (Fig 5). Initial measurements showed that Cs shRNA cells had lower (*p* < 0.05) basal oxygen consumption (Fig 5A and 5C) and higher (*p* < 0.05) proton production (Fig 5B) compared to Con shRNA cells. Subsequent measurements with application of inhibitors and uncouplers revealed that, in comparison to Con shRNA cells, Cs shRNA cells had low (*p* < 0.05) maximal oxygen consumption, spare respiratory capacity (Fig 5C), proton leak and aerobic ATP production, but greater (*p* < 0.05) non-mitochondrial respiration (Fig 5D).

**Fig 6:**
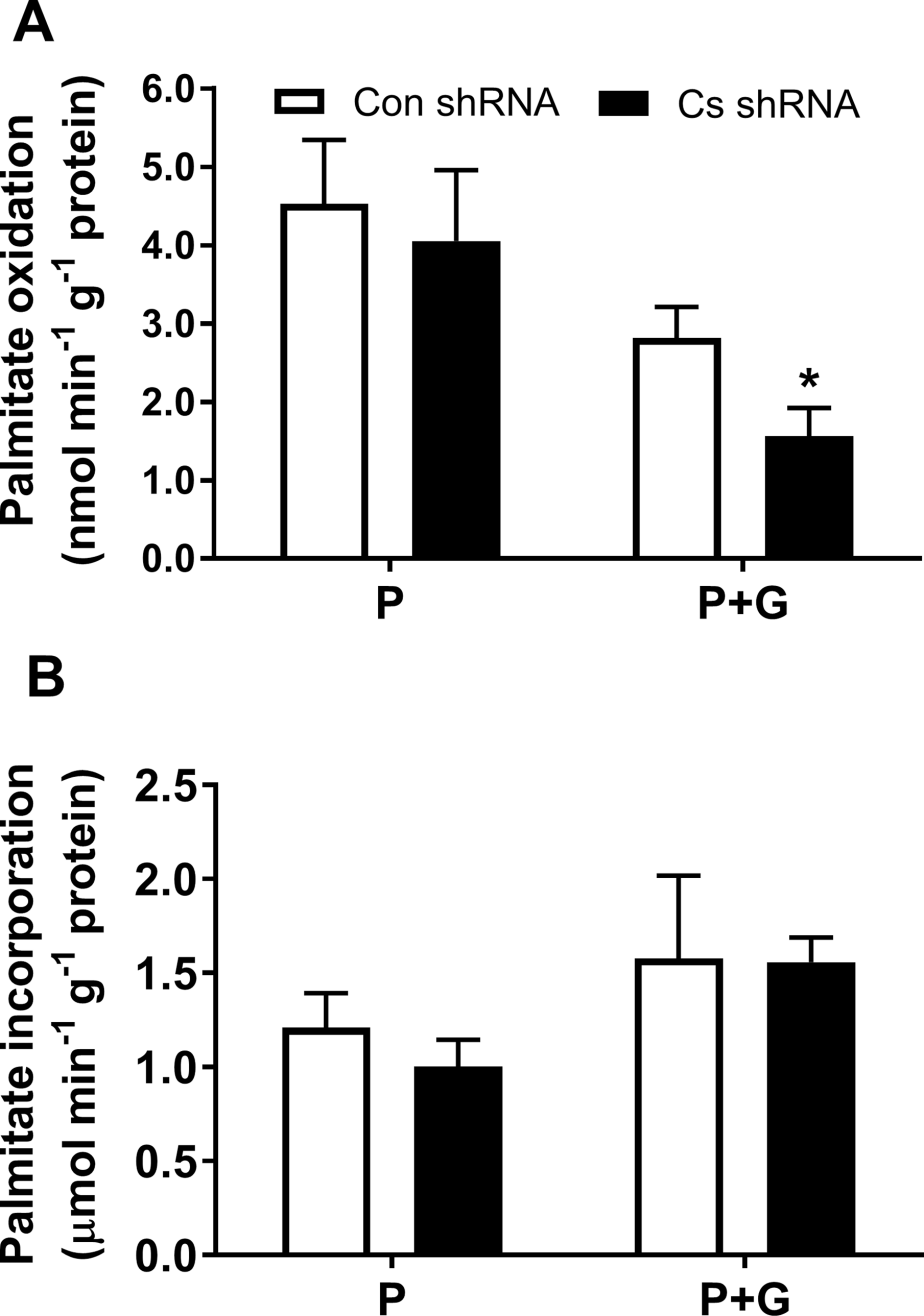
Palmitate metabolism in C2C12 muscle cells. The cells were treated with Con shRNA or Cs shRNA and then incubated in the differentiation media containing 5.5 mM glucose (G) and/or 0.8 mM palmitate for 2 h (P). A, palmitate oxidation was assessed by adding [1-^14^C]palmitate to the media and measuring its incorporation into CO_2_ (n = 6); B, palmitate incorporation was measured as from the amount of [1-^14^C]palmitate cell lysates generated from the cells after the experiments with palmitate oxidation (n = 6). Results are means ± SEM; * *p* < 0.05 between Cs shRNA and Con shRNA cells.

Palmitate metabolism was also assessed in Con shRNA and *Cs* shRNA cells (Fig 6). There was no difference between Con shRNA and *Cs* shRNA cells in palmitate oxidation when these cells were incubated in palmitate without glucose. However, palmitate oxidation was lower (*p* < 0.05) in *Cs* shRNA cells compared to Con shRNA in the presence of 5.5 mM glucose (Fig 6A). There were no significant differences between Con shRNA and Cs shRNA cells in palmitate incorporation which tended to increase when 5.5 mM glucose was present in to the media containing 0.8 mM palmitate (Fig 6B).

**Fig 7:**
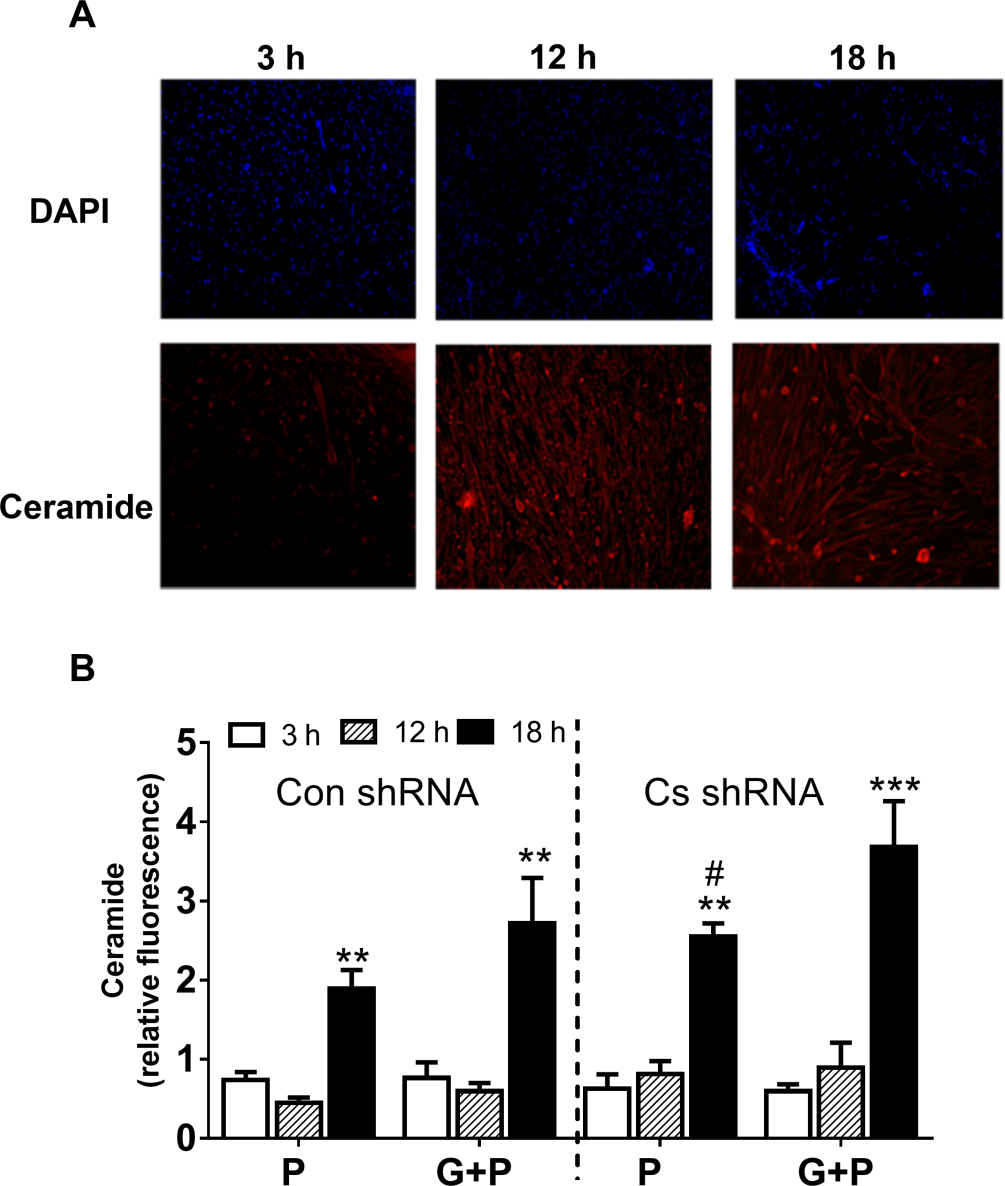
Ceramide accumulation in C2C12 muscle cells. The cells were treated with Con shRNA or Cs shRNA and then incubated in the differentiation media containing 0.8 mM palmitate (P) and/or 0.8 mM palmitate with 5.5 mM glucose for 3, 12 or 18 h (G+P). A, Representative images for DAPI and ceramide staining, respectively; B, Ceramide accumulation which was quantified as relative fluorescence with normalization to background and DAPI staining. Results are means ± SEM (n = 10 each); ** *p* < 0.01, *** *p* < 0.001 between 3 h and 18 h incubation # *p* < 0.05 between Cs shRNA and Con shRNA cells.

Fig 7 shows data on ceramide levels in Con shRNA and *Cs* shRNA cells after incubation in the media containing 0.8 mM palmitate without glucose (P experiments) or with 5.5 mM glucose (P+G experiments). Cellular fluorescence for ceramide was normalised to DAPI fluorescence which reflected nuclear DNA content in this analysis (Fig 7A). There was little change in the ceramide content of the cells from 3 to 12-h incubation independently of the experimental conditions (Fig 7B). However, ceramide accumulation increased (*p* < 0.01–0.001) afterwards and was greater (*p* < 0.05) in P+G compared to P experiments. For P experiments, ceramide levels were greater (*p* < 0.05) in *Cs* shRNA cells compared to Con shRNA cells after 18 h. The same tendency of the difference (*p* = 0.068) between *Cs* shRNA cells and Con shRNA cells was observed in P+G experiments.

## Discussion

The aims of the study were twofold. Firstly, we tested the hypothesis that the A/J variant of the H55N polymorphism is associated with low CS activity. Secondly, we examined if low CS activity interferes with functioning and metabolism of muscle cells. Our results support both these hypotheses. Indeed, mouse strains carrying the A/J variant of H55N polymorphism showed lower CS activity in all tissues measured compared to mouse strains expressing the wild type *Cs* allele. Furthermore, the introduction of this H55N substitution into the *Cs* cDNA caused a reduction in enzyme activity of the recombinant CS protein. In agreement with our second hypothesis, shRNA mediated *Cs* knockdown led to low rates of oxidative phosphorylation, increased reliance on anaerobic glycolysis and a high proliferation rates of C2C12 muscle cells. We have also observed higher rates of ceramide accumulation in muscle cells with low CS activity compared to the control cells during incubation with palmitate. It appears that a 47% reduction in CS activity is associated with alterations in cellular metabolism indicating reduced metabolic flexibility which becomes significant when cells are exposed to substrate rich media containing palmitate.

Firstly, CS activity was measured in different tissues of mice in order to examine the hypothesis concerning the link between H55N polymorphism and CS activity. The results are in agreement with previous findings showing lower CS activity in the gastrocnemius muscles of A/J mice compared to other mouse strains [8,19]. The same phenomenon has now been replicated in the heart and liver with additional data from mice which carry at least one allele containing the A/J variant of *Cs* gene, i.e. B6.A and B6/B6.A mice. Interestingly, however, the variation in CS activity between the strains was greater for the gastrocnemius muscles than for the heart and liver. Skeletal muscle is a malleable tissue which shows differences in fibre type composition and mitochondrial content between homologous skeletal muscles of various mouse strains [12,20]. These differences might have affected CS activity in the gastrocnemius muscle. However, we are unaware of any data showing significant differences between the mouse strains in the mitochondrial content of heart and liver. Interestingly, analyses of data for the heterozygous B6/B6.A animals suggest a dominant effect of the A/J allele on CS activity in all three types of tissues. In summary, our findings provide further evidence in support of the hypothesis that A/J variant of H55N polymorphism causes a reduction in CS activity in diverse tissues of mice.

It is important to note that the congenic region of B6.A strain contains a number of genes that are polymorphic between B6 and A/J strains. In addition to *Cs*, *Shmt2*, *Lrp1*, Zbtb39, *BC089597*, *Gls2*, *Apon*, *Stat2* and *Pan2* carry non-synonymous SNPs between the two strains. Some of those genes (*Shmt2* and *Gls2*) may even play a role in mitochondrial function. Therefore, to eliminate possible confounding effects of other genes we compared the enzymatic activity between the A/J and wildtype variants of *Cs* following the overexpression of the recombinant CS protein in transformed RosettaBlue (DE3) cells. This substitution caused 15–50% reduction in CS activity of the recombinant CS protein. There might be some degradation of CS protein when the overexpression was carried out at 30°C [14,21]. Nevertheless, higher CS activity was consistently observed in the samples generated using B6 construct compared to A/J construct. Hence, the H55N polymorphism of the Cs gene is responsible for the CS activity differences between the B6 and A/J strains.

An inborn defect in the CS enzyme is hypothesized to cause low rates of fatty acid oxidation in the cell cultures generated from muscle biopsies of patients with type 2 diabetes [10,11]. However, no direct experimental evidence was provided in support of this hypothesis. There are likely to be multiple differences between cells of diabetic patients and healthy volunteers and differences in lipid metabolism are not necessarily linked to CS. Furthermore, maximum CS activity exceeds the flux through the Krebs cycle by several orders of magnitude [6]. Thus, a ~20% reduction in insulin stimulated CS activity, as demonstrated by Ortenblad *et al*.[11], might not have a significant effect on cellular metabolism. In our study, shRNA mediated knockdown of CS activity in C2C12 muscle cells provided a good model to test the hypothesis that CS activity is implicit in the control of cellular metabolism. This intervention helped to generate a loss of CS activity which was of a similar magnitude to that of the H55N induced reduction. ~47% knockdown of CS activity was associated with low oxygen consumption and increased proton production by these muscle cells. Indeed, CS knockdown also promotes reliance on anaerobic glycolysis and stimulates proliferation of human cervical carcinoma cells [22]. It appears that a similar phenomenon occurs in C2C12 cells. In the current study, there was also a reduction in citrate levels in cells with CS knockdown. Cytosolic citrate can bind and inhibit phosphofructokinase 1 which is a key enzyme in anaerobic glycolysis [23,24]. Reduced inhibition of phosphofructokinase 1 might act to promote glycolysis and thus suppress fatty acid oxidation. Maximal oxygen uptake, aerobic ATP synthesis and respiratory capacity were also lower in these cells compared to the controls. It appears that ~47% reduction in CS activity might limit the peak rate of aerobic ATP production in muscle cells.

We have also examined effects of low CS activity on mitochondrial markers and signalling proteins including AMPK which is involved in mitochondrial biogenesis. The results of these measurements do not show any differences between C2C12 cells with reduced and normal levels of CS. Thus, reduction in aerobic capacity of C2C12 cells with low CS activity is not due to low mitochondrial content and cells do not experience energy stress when incubated in standard media for cell culturing. It is likely that anaerobic glycolysis can compensate for alterations in aerobic metabolism under the basal conditions when cells are not subjected to high metabolic demands.

A limitation of this study is that the utilised homogenization process abolishes mitochondrial integrity. Thus it is not possible to distinguish between cytosolic and mitochondrial CS activity or citrate accumulation. This should be taken into account when assessing these results. Nevertheless, observations of whole-cell physiological changes and metabolism remain valid.

Muscle cells are often exposed to exercise or the physiological consequences of high fat diet which induces significant stress in these cells. Defective skeletal muscle cell metabolism is seen as a major contributor to the pathology of obesity and insulin resistance, particularly the inability to upregulate lipid oxidation in response to a high glucose and palmitate overload [25].

Prolonged incubation with palmitate, which is the most abundant fatty acid, leads to apoptosis and impairment of mitochondrial function in various types of muscle cells [26,27]. Promotion of palmitate oxidation by overexpression of mitochondrial carnitine palmitoyltransferase 1 can reduce palmitate-induced apoptosis [26]. Indeed, we observed accelerated accumulation of ceramide in muscle cells with low CS activity during incubation with palmitate. This is probably a consequence of the inability of these cells to increase the rate of fatty-acid oxidation in response to a high concentration of palmitate, an indication of metabolic inflexibility [25].

In summary, the H55N polymorphism affects CS activity in tissues of mice carrying A/J variant of Cs gene. Low activity of CS results in an impairment in aerobic ATP synthesis and low rates of fatty acid oxidation in muscle cells. These alterations in lipid metabolism render the muscle cell less resistant to metabolic stress as induced by high extracellular levels of palmitate.

## Supporting information

S1 Table Supporting data for Fig 1A. (PDF)

S2 Table Supporting data for Fig 1B. (PDF)

S3 Table Supporting data for Fig 1C. (PDF)

S4 Table Supporting data for Fig 2C. (PDF)

S5 Table Supporting data for Fig 3A. (PDF)

S6 Table Supporting data for Fig 3B. (PDF)

S7 Table Supporting data for Fig 3C. (PDF)

S8 Table Supporting data for Fig 3D. (PDF)

S9 Table Supporting data for Fig 3E. (PDF)

S10 Table Supporting data for Fig 4A. (PDF)

S11 Table Supporting data for Fig 4B. (PDF)

S12 Table Supporting data for Fig 4C. (PDF)

S13 Table Supporting data for Fig 4D. (PDF)

S14 Table Supporting data for Fig 5A. (PDF)

S15 Table Supporting data for Fig 5B. (PDF)

S16 Table Supporting data for Fig 5C. (PDF)

S17 Table Supporting data for Fig 5D. (PDF)

S18 Table Supporting data for Fig 6A. (PDF)

S19 Table Supporting data for Fig 6B. (PDF)

S20 Table Supporting data for Fig 7B. (PDF)
