# Supplementary material for: H55N polymorphism is associated with low citrate synthase activity which regulates lipid metabolism in mouse muscle cells
Source: PLoS One. 2017 Nov 2;12(11):e0185789. doi: 10.1371/journal.pone.0185789 (PMC5667803; doi:10.1371/journal.pone.0185789)
Supplement: S1 Table — (PDF) [file pone.0185789.s001.pdf]

S1 Table. Supporting data for Fig. 1A.

Samples	Balb	B6	B6/B.A	B6.A	A/J
1	3271	3366	2225	2998	1709
2	3174	4070	1936	2401	2086
3	3469	2379	2169	2318	1386
4	3533	3222	2295	2273	2344
5	3281	3294	2525	1778	1578
6	3417	3048	2635	2446	2042
7		3605	1881	1408	
8		4852	1988	1688	
9		4316	2527	1492	
10		3442		2896	
11		2691		1896	

12		2246		1271	
13		2236		1460	
