# Supplementary material for: H55N polymorphism is associated with low citrate synthase activity which regulates lipid metabolism in mouse muscle cells
Source: PLoS One. 2017 Nov 2;12(11):e0185789. doi: 10.1371/journal.pone.0185789 (PMC5667803; doi:10.1371/journal.pone.0185789)
Supplement: S2 Table — (PDF) [file pone.0185789.s002.pdf]

S2 Table. Supporting data for Fig. 1B.

Samples	Balb	B6	B6/B6.A	B6.A	A/J
**1**	241	354	223	173	224
**2**	277	239	204	162	170
**3**	371	301	202	137	192
**4**	341	308	175	185	149
**5**	320	324	238	210	199
**6**	265	337	235	222	228
**7**		241	241	216	
**8**		185	197	194	
**9**		185	183	264	
**10**		245		244	
**11**		354		215	

**12**		272		197	
**13**		311		205	
