# Supplementary material for: H55N polymorphism is associated with low citrate synthase activity which regulates lipid metabolism in mouse muscle cells
Source: PLoS One. 2017 Nov 2;12(11):e0185789. doi: 10.1371/journal.pone.0185789 (PMC5667803; doi:10.1371/journal.pone.0185789)
Supplement: S3 Table — (PDF) [file pone.0185789.s003.pdf]

S3 Table. Supporting data for Fig. 1C.

Samples	Balb	B6	B6/B6.A	B6.A	A/J
**1**	410	809	400	306	249
**2**	893	696	581	346	293
**3**		616	346	344	264
**4**	643	610	577	350	388
**5**	644	478	540	382	197
**6**	835	608	461	330	244
**7**	839	686	388	385	233
**8**		553	463	696	
**9**		352	459	403	
**10**		387		660	
**11**		538		356	
**12**		568		353	
**13**		723		352	
