# Supplementary material for: H55N polymorphism is associated with low citrate synthase activity which regulates lipid metabolism in mouse muscle cells
Source: PLoS One. 2017 Nov 2;12(11):e0185789. doi: 10.1371/journal.pone.0185789 (PMC5667803; doi:10.1371/journal.pone.0185789)
Supplement: S4 Table — (PDF) [file pone.0185789.s004.pdf]

S4 Table. Supporting data for Fig. 2C.

B6 construct

**Samples:**	**1**	**2**	**3**	**4**
**Fraction 1**	312	281	216	228
**Fraction 2**	165	189	190	179
**Fraction 3**	102	123	165	148
**Fraction 4**	41	87	107	33
**Fraction 5**	18	14	23	3

A/J construct

**Samples:**	**1**	**2**	**3**	**4**
**Fraction 1**	292	204	193	172
**Fraction 2**	250	133	155	77
**Fraction 3**	117	108	51	11
**Fraction 4**	40	69	24	2
**Fraction 5**	2	28	8	2
