# Supplementary material for: H55N polymorphism is associated with low citrate synthase activity which regulates lipid metabolism in mouse muscle cells
Source: PLoS One. 2017 Nov 2;12(11):e0185789. doi: 10.1371/journal.pone.0185789 (PMC5667803; doi:10.1371/journal.pone.0185789)
Supplement: S5 Table — (PDF) [file pone.0185789.s005.pdf]

S5 Table. Supporting data for Fig. 3A.

Samples:	Con shRNA	Cs shRNA
**1**	246	120
**2**	326	152
**3**	370	153
**4**	270	115
**5**	335	145
**6**	400	177
**7**	427	143
**8**	348	155
**9**	266	128
