# Supplementary material for: H55N polymorphism is associated with low citrate synthase activity which regulates lipid metabolism in mouse muscle cells
Source: PLoS One. 2017 Nov 2;12(11):e0185789. doi: 10.1371/journal.pone.0185789 (PMC5667803; doi:10.1371/journal.pone.0185789)
Supplement: S6 Table — (PDF) [file pone.0185789.s006.pdf]

S6 Table. Supporting data for Fig. 3B.

**Samples:**	**Con shRNA**	**Cs shRNA**
**1**	0.068	0.081
**2**	0.087	0.137
**3**	0.191	0.143
**4**	0.337	0.096
**5**	0.322	0.114
**6**	0.268	0.083
**7**	0.302	0.034
**8**	0.127	0.018
**9**	0.234	0.016
