# Supplementary material for: H55N polymorphism is associated with low citrate synthase activity which regulates lipid metabolism in mouse muscle cells
Source: PLoS One. 2017 Nov 2;12(11):e0185789. doi: 10.1371/journal.pone.0185789 (PMC5667803; doi:10.1371/journal.pone.0185789)
Supplement: S7 Table — (PDF) [file pone.0185789.s007.pdf]

S7 Table. Supporting data for Fig. 3C.

**Samples:**	**Con shRNA**	**Cs shRNA**
**1**	3.23	3.88
**2**	5.81	6.13
**3**	6.53	6.06
**4**	5.39	5.30
**5**	6.36	5.62
**6**	7.41	6.08
**7**	7.97	5.49
**8**	7.34	7.69
**9**	5.13	5.81
