# Supplementary material for: H55N polymorphism is associated with low citrate synthase activity which regulates lipid metabolism in mouse muscle cells
Source: PLoS One. 2017 Nov 2;12(11):e0185789. doi: 10.1371/journal.pone.0185789 (PMC5667803; doi:10.1371/journal.pone.0185789)
Supplement: S9 Table — (PDF) [file pone.0185789.s009.pdf]

S9 Table. Supporting data for Fig. 3E

**Days**	**Con shRNA**				**Days**	**Cs shRNA**			
**0**	0	0	0	0	**0**	0	0	0	0
**0.5**	0.3905	0.2676	0.2297	0.2495	**0.5**	0.4298	0.3089	0.2945	0.6409
**1**	0.4735	0.29	0.2541	0.3245	**1**	0.5028	0.3501	0.3773	0.9614
**1.5**	0.5303	0.2797	0.302	0.3687	**1.5**	0.7692	0.4646	0.4722	1.3941
**2**	0.7606	0.3713	0.4238	0.516	**2**	1.4621	0.7099	0.7321	1.5214
**2.5**	1.2968	0.8332	0.7313	0.7865	**2.5**	2.2737	1.1865	1.3796	2.8051
**3**	2.2069	1.4621	1.2956	1.5204	**3**	3.6107	2.0611	2.4734	3.6483
**3.5**	2.5151	1.852	1.7561	1.8827	**3.5**	3.7987	2.5597	2.8689	3.3208
**4**	3.8981	3.0385	2.9285	3.182	**4**	5.0792	4.3654	4.8448	3.6631
**4.5**	3.6694	3.2092	3.3589	2.9928	**4.5**	4.0801	3.8902	4.1181	2.8863
**5**	4.237	4.1993	4.486	3.7833	**5**	4.4633	4.5655	4.7383	3.2805
**5.5**	3.5481	3.4889	3.8653	3.1638	**5.5**	3.5443	3.6794	3.839	2.8256
**6**	3.8583	3.9062	4.1474	3.7171	**6**	3.7581	4.0745	4.0472	3.2121
**6.5**	3.1586	3.1821	3.4056	2.9746	**6.5**	3.1122	3.3333	3.3584	2.9298
**7**	3.541	3.3427	3.846	3.2216	**7**	3.5983	3.623	3.5931	3.6169

**7.5**	3.0646	3.206	3.3347	3.0872	**7.5**	3.1561	3.2563	3.0395	3.0925
**8**	3.3071	3.5622	3.4518	3.5668	**8**	3.7875	3.4667	3.314	3.5678
**8.33**	3.2285	3.4081	3.4096	3.35	**8.33**	3.2585	3.2132	3.0279	3.0526
