# Supplementary material for: H55N polymorphism is associated with low citrate synthase activity which regulates lipid metabolism in mouse muscle cells
Source: PLoS One. 2017 Nov 2;12(11):e0185789. doi: 10.1371/journal.pone.0185789 (PMC5667803; doi:10.1371/journal.pone.0185789)
Supplement: S10 Table — (PDF) [file pone.0185789.s010.pdf]

S10 Table. Supporting data for Fig. 4A

	**P-AMPK/AMPK**	
**Samples**	**Con shRNA**	**Cs shRNA**
**1**	1.55	1.16
**2**	1.43	1.37
**3**	1.07	1.28
**4**	0.96	0.79
**5**	1.08	0.92
**6**	1.05	0.78
**7**	0.96	0.59
**8**	0.80	0.69
