# Supplementary material for: H55N polymorphism is associated with low citrate synthase activity which regulates lipid metabolism in mouse muscle cells
Source: PLoS One. 2017 Nov 2;12(11):e0185789. doi: 10.1371/journal.pone.0185789 (PMC5667803; doi:10.1371/journal.pone.0185789)
Supplement: S11 Table — (PDF) [file pone.0185789.s011.pdf]

S11 Table. Supporting data for Fig. 4B

	P-ACC/ACC	
**Samples**	**Con shRNA**	**Cs shRNA**
**1**	1.98	1.76
**2**	1.65	1.81
**3**	1.45	2.32
**4**	3.16	5.24
**5**	3.31	5.00
**6**	2.79	2.02
**7**	2.84	1.74
**8**	3.24	3.17
