# Supplementary material for: H55N polymorphism is associated with low citrate synthase activity which regulates lipid metabolism in mouse muscle cells
Source: PLoS One. 2017 Nov 2;12(11):e0185789. doi: 10.1371/journal.pone.0185789 (PMC5667803; doi:10.1371/journal.pone.0185789)
Supplement: S12 Table — (PDF) [file pone.0185789.s012.pdf]

S12 Table. Supporting data for Fig. 4C

	**P-p38/p38**	
**Samples**	**Con shRNA**	**Cs shRNA**
**1**	0.619	0.698
**2**	0.612	0.743
**3**	0.576	1.418
**4**	1.366	0.618
**5**	0.773	0.687
**6**	0.577	0.869
**7**	0.916	0.881
**8**	0.788	0.948
