# Supplementary material for: H55N polymorphism is associated with low citrate synthase activity which regulates lipid metabolism in mouse muscle cells
Source: PLoS One. 2017 Nov 2;12(11):e0185789. doi: 10.1371/journal.pone.0185789 (PMC5667803; doi:10.1371/journal.pone.0185789)
Supplement: S13 Table — (PDF) [file pone.0185789.s013.pdf]

S13 Table. Supporting data for Fig. 4D

	**P-mTOR/mTOR**	
**Samples**	**Con shRNA**	**Cs shRNA**
**1**	1.85	1.14
**2**	1.70	1.13
**3**	1.03	0.68
**4**	0.70	0.92
**5**	0.83	1.29
**6**	0.98	1.08
**7**	1.11	1.43
**8**	1.46	0.40
