# Supplementary material for: H55N polymorphism is associated with low citrate synthase activity which regulates lipid metabolism in mouse muscle cells
Source: PLoS One. 2017 Nov 2;12(11):e0185789. doi: 10.1371/journal.pone.0185789 (PMC5667803; doi:10.1371/journal.pone.0185789)
Supplement: S14 Table — (PDF) [file pone.0185789.s014.pdf]

S14 Table. Supporting data for Fig. 5A

Con shRNA cells

Time (min)	1	2	3	4	5	6	7	8	9	10
**9**	215	318	503	296	276	240	143	298	272	305
**18**	248	343	616	304	285	243	112	293	290	328
**27**	255	367	583	296	305	259	110	307	300	297
**36**	100	116	314	100	150	136	99	96	104	124
**45**	116	153	358	182	186	148	91	111	144	155
**54**	128	160	369	207	198	136	75	108	135	134
**63**	519	488	846	539	613	459	301	499	562	621
**72**	459	424	807	429	490	619	297	414	528	574
**81**	366	314	677	369	408	492	227	300	414	460
**90**	37	22	241	79	51	60	16	18	45	32
**99**	48	50	236	107	63	73	35	11	37	52
**108**	81	79	261	131	82	77	59	35	67	64

S14 Table. Supporting data for Fig. 5A (continued)

Cs shRNA cells:

Time (min)	1	2	3	4	5	6	7	8	9	10
**9**	155	153	100	207	150	222	274	223	302	223
**18**	154	181	169	194	175	263	289	371	427	231
**27**	162	178	187	206	170	261	299	354	379	224
**36**	32	38	144	141	146	154	185	212	235	70
**45**	57	73	146	165	158	157	184	233	256	90
**54**	66	64	146	190	153	153	174	211	246	90
**63**	26	212	136	766	316	538	685	611	513	548
**72**	26	180	127	696	293	397	565	596	464	444
**81**	25	145	115	596	250	339	498	517	386	363
**90**	0	11	99	62	113	116	188	116	191	41
**99**	8	32	113	71	117	112	174	121	192	43

**108**	31	35	146	99	136	127	182	137	217	67
