# Supplementary material for: H55N polymorphism is associated with low citrate synthase activity which regulates lipid metabolism in mouse muscle cells
Source: PLoS One. 2017 Nov 2;12(11):e0185789. doi: 10.1371/journal.pone.0185789 (PMC5667803; doi:10.1371/journal.pone.0185789)
Supplement: S15 Table — (PDF) [file pone.0185789.s015.pdf]

S15 Table. Supporting data for Fig. 5B

Con shRNA cells:

Time (min)	1	2	3	4	5	6	7	8	9	10
**9**	180	147	192	124	167	116	186	112	203	245
**18**	172	146	172	123	173	113	184	109	200	237
**27**	165	150	172	125	158	120	180	106	182	223
**36**	235	219	355	230	248	217	320	237	329	382
**45**	251	238	372	237	253	239	337	231	340	395
**54**	241	226	366	236	247	242	322	242	354	396
**63**	228	225	293	205	402	216	306	228	360	389
**72**	232	228	1348	198	386	212	281	229	353	372
**81**	231	218	1331	195	403	203	275	226	330	348
**90**	186	186	1165	172	246	179	280	209	270	314
**99**	212	196	1151	236	222	196	283	227	295	317
**108**	191	189	1109	185	224	199	252	212	281	303

Cs shRNA cells:

Time (min)	1	2	3	4	5	6	7	8	9	10
**9**	337	180	332	260	150	194	221	250	257	261
**18**	338	186	404	310	170	195	223	251	247	275
**27**	348	192	397	248	152	201	222	256	237	271
**36**	341	238	312	385	176	353	362	270	312	372
**45**	417	235	317	403	207	373	375	305	327	372
**54**	401	227	313	365	206	361	363	307	358	385

**63**	357	248	263	419	285	370	439	321	508	856
**72**	362	260	282	402	219	363	567	319	534	875
**81**	365	250	265	384	211	349	518	299	506	857
**90**	336	268	278	359	182	420	437	279	469	865
**99**	398	274	264	390	204	447	477	315	541	856
**108**	347	280	256	396	198	391	436	322	506	833
