# Supplementary material for: H55N polymorphism is associated with low citrate synthase activity which regulates lipid metabolism in mouse muscle cells
Source: PLoS One. 2017 Nov 2;12(11):e0185789. doi: 10.1371/journal.pone.0185789 (PMC5667803; doi:10.1371/journal.pone.0185789)
Supplement: S16 Table — (PDF) [file pone.0185789.s016.pdf]

**S16 Table. Supporting data for Fig. 5C**

**Con shRNA cells**

| | **1** | **2** | **3** | **4** | **5** | **6** | **7** | **8** | **9** | **10** |
|---|---|---|---|---|---|---|---|---|---|---|
| **Basal Rate** | 184 | 292 | 321 | 193 | 223 | 178 | 85 | 278 | 238 | 261 |
| **Maximal Respiration Rate** | 393 | 358 | 530 | 340 | 438 | 454 | 238 | 383 | 452 | 503 |
| **Spare Respiratory Capacity** | 16 | 80 | 27 | 165 | 307 | 401 | 450 | 254 | 401 | 242 |

**Cs shRNA cells**

| | **1** | **2** | **3** | **4** | **5** | **6** | **7** | **8** | **9** | **10** |
|---|---|---|---|---|---|---|---|---|---|---|
| **Basal Rate** | 144 | 145 | 33 | 125 | 43 | 131 | 106 | 192 | 169 | 176 |
| **Maximal Respiration Rate** | 16 | 80 | 27 | 165 | 307 | 401 | 450 | 254 | 401 | 0 |
| **Spare Respiratory Capacity** | 8 | 122 | 176 | 85 | 0 | 0 | 0 | 0 | 0 | 0 |
