# Supplementary material for: H55N polymorphism is associated with low citrate synthase activity which regulates lipid metabolism in mouse muscle cells
Source: PLoS One. 2017 Nov 2;12(11):e0185789. doi: 10.1371/journal.pone.0185789 (PMC5667803; doi:10.1371/journal.pone.0185789)
Supplement: S17 Table — (PDF) [file pone.0185789.s017.pdf]

S17 Table. Supporting data for Fig. 5D

Con shRNA cells

	**1**	**2**	**3**	**4**	**5**	**6**	**7**	**8**	**9**	**10**
**Proton Leak**	59	93	101	57	113	70	52	84	78	89
**ATP Production**	125	199	221	136	111	108	34	194	160	172
**Non-mitochondrial respiration**	55	50	0	106	65	70	37	21	50	49

Cs shRNA cells

	**1**	**2**	**3**	**4**	**5**	**6**	**7**	**8**	**9**	**10**
**Proton Leak**	39	32	27	88	30	37	7	94	45	33
**ATP Production**	105	113	7	37	13	94	99	97	124	142
**Non-mitochondrial respiration**	13	26	119	78	122	118	181	125	200	50
