# Supplementary material for: H55N polymorphism is associated with low citrate synthase activity which regulates lipid metabolism in mouse muscle cells
Source: PLoS One. 2017 Nov 2;12(11):e0185789. doi: 10.1371/journal.pone.0185789 (PMC5667803; doi:10.1371/journal.pone.0185789)
Supplement: S18 Table — (PDF) [file pone.0185789.s018.pdf]

S18 Table. Supporting data for Fig. 6A

Con shRNA cells

	**1**	2	**3**	4	**5**	6
**P**	2.75	7.32	3.33	3.24	6.84	3.66
**G+P**	2.44	4.16	2.05	2.24	3.95	2.06

Cs shRNA cells

	**1**	2	**3**	4	**5**	6
**P**	2.24	7.74	2.39	3.78	5.63	2.52
**G+P**	0.63	2.70	1.21	1.39	2.59	0.85
