# Supplementary material for: H55N polymorphism is associated with low citrate synthase activity which regulates lipid metabolism in mouse muscle cells
Source: PLoS One. 2017 Nov 2;12(11):e0185789. doi: 10.1371/journal.pone.0185789 (PMC5667803; doi:10.1371/journal.pone.0185789)
Supplement: S19 Table — (PDF) [file pone.0185789.s019.pdf]

S19 Table. Supporting data for Fig. 6B

Con shRNA cells

	**1**	**2**	**3**	**4**	**5**	**6**
**P**	0.86	1.77	1.02	0.80	1.79	1.02
**P+G**	1.24	3.20	1.84	1.11	0.00	2.08

Cs shRNA cells

	**1**	**2**	**3**	**4**	**5**	**6**
**P**	0.71	1.57	0.89	0.67	1.25	0.94
**G+P**	1.15	1.71	1.79	1.16	1.87	1.67
