# Supplementary material for: H55N polymorphism is associated with low citrate synthase activity which regulates lipid metabolism in mouse muscle cells
Source: PLoS One. 2017 Nov 2;12(11):e0185789. doi: 10.1371/journal.pone.0185789 (PMC5667803; doi:10.1371/journal.pone.0185789)
Supplement: S20 Table — (PDF) [file pone.0185789.s020.pdf]

S20 Table. Supporting data for Fig. 7B

3 hours after incubation

**Con shRNA:**	**1**	**2**	**3**	**4**	**5**	**6**	**7**	**8**	**9**
**P**	0.74	0.61	1.02	0.44	0.90	0.53	1.24	0.28	0.86
**G+P**	0.60	0.83	0.98	0.52	0.23	0.23	0.71	2.19	0.57
**Cs shRNA:**	**1**	**2**	**3**	**4**	**5**	**6**	**7**	**8**	**9**
**P**	0.02	0.48	0.42	0.24	0.98	0.57	1.81	0.23	0.90
**G+P**	0.34	0.48	0.72	0.53	0.75	0.47	0.77	0.20	1.08

12 hours after incubation

**Con shRNA:**	**1**	**2**	**3**	**4**	**5**	**6**	**7**	**8**	**9**
**P**	0.43	0.45	0.12	0.37	0.39	0.81	0.63	0.50	0.35
**G+P**	0.68	1.24	0.45	0.29	0.84	0.70	0.54	0.24	0.35
**Cs shRNA:**	**1**	**2**	**3**	**4**	**5**	**6**	**7**	**8**	**9**
**P**	1.96	1.16	0.58	0.23	0.51	0.63	0.75	0.58	0.88
**G+P**	0.33	1.24	1.15	0.27	0.19	0.43	3.20	0.91	0.30
